# Review of Salvage Therapy for Biochemically Recurrent Prostate Cancer: The Role of Imaging and Rationale for Systemic Salvage Targeted Anti-Prostate-Specific Membrane Antigen Radioimmunotherapy

**DOI:** 10.1155/2012/921674

**Published:** 2012-05-28

**Authors:** Satyajit Kosuri, Naveed H. Akhtar, Michael Smith, Joseph R. Osborne, Scott T. Tagawa

**Affiliations:** ^1^Division of Hematology and Medical Oncology, Department of Medicine, Weill Cornell Medical College, New York, NY 10065, USA; ^2^Department of Radiation Oncology, Weill Cornell Medical College, New York, NY 10065, USA; ^3^Division of Nuclear Medicine, Department of Radiology, Weill Cornell Medical College, New York, NY 10065, USA; ^4^Weill Cornell Cancer Center, New York, NY 10065, USA; ^5^Department of Urology, Weill Cornell Medical College, New York, NY 10065, USA

## Abstract

Despite local therapy with curative intent, approximately 30% of men suffer from biochemical relapse. Though some of these PSA relapses are not life threatening, many men eventually progress to metastatic disease and die of prostate cancer. Local therapy is an option for some men, but many have progression of disease following local salvage attempts. One significant issue in this setting is the lack of reliable imaging biomarkers to guide the use of local salvage therapy, as the likely reason for a low cure rate is the presence of undetected micrometastatic disease outside of the prostate/prostate bed. Androgen deprivation therapy is a cornerstone of therapy in the salvage setting. While subsets may benefit in terms of delay in time to metastatic disease and/or death, research is ongoing to improve salvage systemic therapy. Prostate-specific membrane antigen (PSMA) is highly overexpressed by the majority of prostate cancers. While initial methods of exploiting PSMA's high and selective expression were suboptimal, additional work in both imaging and therapeutics is progressing. Salvage therapy and imaging modalities in this setting are briefly reviewed, and the rationale for PSMA-based systemic salvage radioimmunotherapy is described.

## 1. Prostate-Specific Antigen and Biochemical Relapse

Clinically localized prostate cancer (PC) may have a variable, often protracted course from first diagnosis to metastasis [1, 2]. Despite recent controversies, prostate-specific antigen (PSA) has not only revolutionized diagnosis but is also used to monitor disease recurrence after primary treatment options such as radical prostatectomy (RP) or local definitive radiotherapy (RT). An important aspect of monitoring is the concept of biochemical recurrence (BCR) which can be defined within the framework of PSA. A primary definition had proven elusive as there are considerable differences between the primary therapies in regards to their PSA kinetics [3]. Following prostatectomy, absolute PSA values of 0.2–0.4 ng/mL are commonly used to define BCR, with a PSA of 0.4 ng/mL followed by another increase suggested for inclusion in clinical trials for men with BCR following RP [4, 5]. In the post-RT setting, an increase of 2 ng/mL from the patients' post-RT nadir is used as the marker for recurrent/persistent disease (biochemical failure) [6].

In many parts of the world, the majority of men diagnosed with PC are usually well suited for local curative attempts with RP or RT. In this population it has been shown that BCR occurs in 12–42% [7] and 22–69% [8], respectively, overall approximating 30% of patients treated with local therapy for curative intent [5, 9, 10]. In the United States alone, it is estimated that approximately 50,000 patients are diagnosed with BCR annually [4, 11].

## 2. Salvage Therapy: Local Options

Once these patients experience BCR, the decision to start secondary or salvage therapy is a process for which may be as complicated as the decision about primary therapy. As at initial diagnosis, the range of outcomes after BCR is variable, with some men progressing to overt metastatic disease and death despite therapy and others dying of other causes even without further PC intervention [12]. As a concept akin to other solid tumors, those with local recurrence might be cured with local therapy; some with systemic recurrence may benefit from systemic therapy, though as with other solid tumors in general, only those with local recurrence tend to be cured with salvage therapy. There are many options that include salvage RP, brachytherapy, external beam radiation therapy, cryotherapy, androgen deprivation therapy (ADT), or a combination of these modalities.

For those with BCR following radiation therapy, salvage radical prostatectomy (SRP) after primary radiotherapy can offer an effective management option. Eastham and colleagues studied 146 patients who underwent SRP for biopsy-proven local recurrence of PC [13]. In this study BCR was defined as a serum PSA of 0.2 ng/mL or higher or the initiation of androgen deprivation therapy after radiotherapy. Over a period of 5 years the recurrence-free probability was 54%, and only one patient experienced a clinical local recurrence, with a 5-year cumulative incidence of death from PC of 4%. As all of the prior reported experience was retrospective, the Cancer and Leukemia Group B (CALGB) performed a multicenter prospective study of SRP in patients who had BCR after radiotherapy. In this study of 41 patients, the 5-year biochemical-free survival was 55% and overall survival (OS) was 85% [14]. The time to first incontinent-free rates at 3, 6, and 12 months after surgery were 90%, 18%, and 9%, and time to first erectile dysfunction-free rates following SRP at 3, 6, and 12 months were 87%, 25%, and 14%. Despite these potentially encouraging efficacy results, SP is currently reserved for a highly select population based upon a number of factors, including real and/or perceived toxicity.

Salvage cryotherapy is an option which some see as less invasive approach to surgery with fewer side effects in the absence of prospective randomized studies. A retrospective analysis examined 76 patients over a 10-year period with a mean Gleason score of 7, who had prostate cryotherapy as salvage therapy before January 1999. At the end of this study, 43 of 76 men (56.6%) were still alive; 33 men (43.4%) had died but only 13.2% from prostate cancer and 22.4% from noncancerous causes, and 6.6% died from unknown causes [15]. A pooled analysis of salvage cryoablation demonstrated 54.5% 5-year actuarial biochemical disease-free survival with an incontinence rate of 4.4% and rectal fistula rate of 1.2% [16]. These and other investigators have concluded that cryosurgery is safe and effective treatment in selected patients in whom radiation therapy fails [15–17]. Further study is necessary, including improvement and standardization of technique.

One option commonly offered to patients with BCR after primary RP is salvage radiation therapy (SRT). Most of the available data comes from retrospective series. Stephenson et al. analyzed data from 17 tertiary care centers, evaluating 1540 patients. The six-year progression-free probability was 32% overall, 48% for patients with a pre-SRT PSA less than or equal to 0.5, 40% with a PSA > 0.5–1, 28% for patients with a PSA 1–1.5, and 18% for PSA greater than 1.5. These findings suggest that delivering SRT at the earliest sign of recurrence, when the PSA is low, is optimal, as nearly half of patients may have a long-term PSA response, including some with other unfavorable prognostic factors, including a PSA doubling time of 10 months or less or with poorly differentiated (Gleason 8–10) histology. A nomogram is available utilizing independently significant variables, including PSA level before SRT, prostatectomy Gleason score, PSA doubling time, surgical margins, androgen-deprivation therapy before or during RT therapy, and lymph node metastasis [18].

A retrospective review from Johns Hopkins included 635 men who previously underwent RP and were subsequently observed (63%), underwent SRT (25%), or SRT + hormonal therapy (12%) for either a biochemical or local recurrence. SRT was associated with a threefold increase in prostate cancer-specific survival (CSS) compared to those not treated with SRT (HR 0.32, *P* < 0.001). The addition of hormonal therapy did not improve CSS. Without long-term followup this benefit in CSS was limited to those with a doubling time of less than 6 months and persisted after adjustment for other prognostic factors. SRT delivered greater than two years after recurrence or, for those men whose PSA never became undetectable after RP, did not result in improvement in CSS at the time of analysis [19].

Although there are limitations in the evaluation of retrospective data, these reports provide solid evidence for the benefit of early SRT. Important factors to consider in determining the need for SRT include preoperative and pre-RT PSA, postrecurrence doubling time, pathologic features suggestive of a local recurrence (e.g., positive margins), achievement or nonachievement of a nondetectable PSA post-operatively, pattern of rise of PSA (whether or not consistent with a local recurrence), long recurrence interval from surgery, as well as patient factors [18, 20, 21].

## 3. Imaging in the Setting of Biochemical Relapse

One of the major issues with local therapy (whether for newly diagnosed clinically localized disease or in the setting of BCR) is the lack of ability to accurately determine the presence or absence of distant metastatic disease. It is likely that the most significant reason for failure of most attempts at salvage therapy for biochemically recurrent PC is the presence of undetected metastatic disease. Conventional imaging techniques such as transrectal ultrasonography, magnetic resonance imaging (MRI), computed tomography (CT), and ^99^Tm-MDP scintigraphy (bone scan) are usually not sensitive or specific enough to detect metastatic or recurrent prostate disease [22–28]. Therefore, an increase in PSA may precede a clinically detectable recurrent pelvic or metastatic cancer by months to years [29].

Though initial attempts using monoclonal antibodies (mAbs) to PSA and PAP were unsuccessful [30], more recently various and more specific markers of PC have been identified, including cell surface proteins, glycoprotein, receptors, enzymes, and peptides [31]. Prostate-specific membrane antigen (PSMA) is the most well established, highly specific prostate epithelial cell membrane antigen known [32–36]. The first and only approved agent for targeting PSMA in PC is ^111^In-capromab [37].

An initial study utilizing capromab pendetide in men BCR after prostatectomy and lymphadenectomy demonstrated safety [38]. Kahn et al. performed a study in 32 men with BCR after prostatectomy prior to SRT; 61% of those with evidence of local disease only had a durable response to SRT versus 28% with durable response if they had evidence of distant disease on ^111^In-capromab imaging [39]. However, while additional similar studies support these results [40], others have demonstrated no benefit with the use of capromab pendetide in selection of patients for local salvage therapy [41, 42]. Some efforts to improve ^111^In-capromab imaging have added SPECT/CT fusion imaging, but results remain suboptimal [43–45].

**Figure 1:**
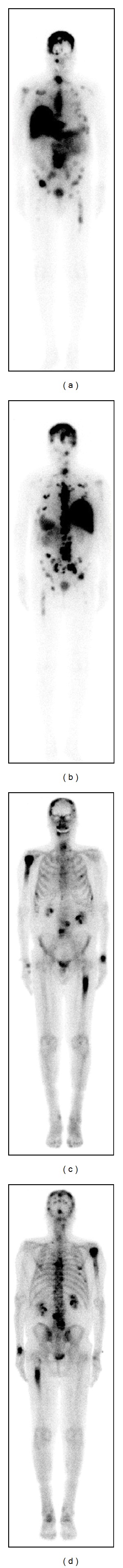
Anterior (a) and posterior (b) planar gamma camera images of radiolabeled J591. A greater number of lesions are apparent compared to anterior (c) and posterior (d) ^99m^Tc-MDP bone scan. Hepatic clearance of radiolabeled mAb results in nonspecific uptake in the liver.

 A major reason for the suboptimal results with capromab pendetide lies with its targeting of the internal domain of PSMA, leading to the inability to bind to viable cells [32–35, 46]. Recognition of these features led to the development of mAbs by Bander et al. to the exposed, extracellular domain of PSMA [46–48]. J591, a deimmunized mAb against the extracellular domain of PSMA, has been the lead clinical candidate [48, 49]. While no formal prostate imaging studies of J591 have been conducted, several therapeutic studies examining the clinical utility of radiolabeled J591 have been performed with built-in imaging components [49–51]. Radiolabeled J591 has successfully targeted (imaged) 89–100% osseous targeting and 69–100% soft tissue targeting [49–51], including cases where J591 demonstrated lesions that were not apparent on the bone scan but were identified on subsequent MR or conventional imaging as the lesion progressed (Figure 1) [52]. Current imaging work with anti-PSMA mAbs involves immune-PET imaging [53, 54]. Additional studies utilize small molecule inhibitors, including ^123^I-MIP-1072, ^123^I-MIP-1095, ^99m^Tc-MIP-1404, and ^99m^Tc-MIP-1405 [55, 56].

## 4. Systemic Therapy for Biochemical Relapse

The addition of hormonal therapy to primary RT has led to improvements for some men with clinically localized PC, possibly by radiosensitization and/or treating micrometastatic disease. This might be true with SRT as well, with several retrospective studies supporting this concept [57, 58]. Initial results of a large, prospective randomized study, RTOG 9601, in which SRT was compared with SRT + bicalutamide in patients with an elevated PSA after prostatectomy have been presented [57]. With a median followup of seven years, a statistically significant improvement in freedom from PSA progression with adjuvant bicalutamide versus RT alone has been reported (57 versus 40%) as well as incidence of metastatic disease (7 versus 13%). RTOG 0534, a Phase III Trial of short-term androgen deprivation with pelvic lymph node or prostate bed only radiotherapy (SPPORT) in PC patients with a rising PSA after RP, is currently accruing (http://www.clinicaltrials.gov/ct2/show/NCT00567580/). Patients are randomly assigned to one of three arms: prostate bed RT only, prostate bed RT + neoadjuvant and concurrent ADT, or RT to the prostate bed and pelvic lymph nodes with neoadjuvant and concurrent ADT [59]. This study will help address the utility of the addition of ADT to SRT.

 Though good local salvage options exist, not all patients qualify or agree to receive them, and most suffer disease progression despite local salvage therapy, likely because of micrometastatic disease outside of the prostate/prostate bed and pelvis that is not apparent on conventional imaging. Therefore systemic therapy is often employed. The most common management option for BCR after local therapy is ADT. While many studies have demonstrated that ADT does not prolong time to metastases and death in all comers, there are subgroups that likely benefit. Higher-grade disease and poorer PSA kinetics (i.e., short PSA doubling time) may predict improvement in outcome with early ADT [60, 61]. Additional evidence to support early ADT stems from the high-risk clinically localized or locally advanced settings [62–64]. However, while ADT may lead to some improvements, toxicity exists [65–70], and it is not curative in this situation. Chemotherapy is proven to improve survival and patient-reported outcomes in late stage disease but, as in most advanced solid tumors, is not able to overcome bulky disease and leads to cures in that setting [71, 72]. The addition of chemotherapy at an earlier stage has demonstrated a survival benefit in many solid tumors (i.e., neoadjuvant or adjuvant chemotherapy in combination with surgery/radiotherapy), presumably by eradicating micrometastatic sites of disease. We await the results of a study examining the use of chemotherapy in combination with hormonal therapy to treat micrometastatic disease in men with BCR after prostatectomy (http://www.clinicaltrials.gov/ct2/show/NCT00514917/) [73].

## 5. Prostate-Specific Membrane Antigen-Based Radioimmunotherapy

 As discussed above, the concept of systemic therapy to eliminate micrometastatic disease has merit. “Targeted therapy” is designed to deliver agents to malignant cells and spare normal cells. PSMA is an ideal target for prostate cancer, based upon its near universal expression in PC. While the initial observations were that expression was limited to prostate cells, it is now known that there are low levels of expression in other tissues, including brush border of small intestine, renal proximal tubule lumen, and salivary glands. However, levels of expression are greatly increased in prostate cancer (as opposed to benign prostatic epithelial cells) and increase with grade, stage, and hormonal therapy [32–35]. Furthermore, alternative sites with low levels of expression have minimal or no exposure to circulating mAb, as they are protected by basement membranes and their luminal surface site of expression. Several studies have demonstrated the ability of radiolabeled J591 to target and treat metastatic castration-resistant prostate cancer (CRPC).

 Two independent phase I radioimmunotherapy (RIT) trials were performed using Yttrium-90 (^90^Y) or Lutetium-177 (^177^Lu) linked via a DOTA chelate to J591 in patients with metastatic CRPC. These trials defined the MTD and further refined dosimetry, pharmacokinetics, and immunogenicity (HAHA) of the radiolabeled mAb with some efficacy seen [50, 51]. Additional phase I and phase II studies utilizing ^177^Lu-J591 have confirmed the ability of J591 to successfully target various sites of metastatic prostate cancer with the majority of subjects receiving full doses of radiolabeled antibody experiencing PSA declines and some measurable disease responses demonstrated [49, 74, 75]. As expected with radioimmunotherapy in general, dose-limiting toxicity is reversible myelosuppression, with a minority of patients also experiencing mAb-related infusion reactions (without pre-medication) or transient grade 1 transaminitis [49–51, 74–76].

Based on the physical properties of radionuclides, differential responses are expected depending upon radionuclide and tumor properties. ^177^Lu is a low energy *β* emitter best for lesions 1–3 mm in diameter, while the higher *β* energy of ^90^Y is best suited for 28–42 mm lesions [77]. An initial review of J591 RIT validated these properties in the clinical CRPC setting [76]. This leads to the hypothesis that ^177^Lu-J591 should be less effective in the bulky metastatic CRPC setting but may lead to significantly more benefit in a micrometastatic disease setting. Indeed, RIT in general may have a higher impact in the minimal disease setting [78–80].

 Prostate cancer is a radiosensitive disease, and BCR is common. Salvage local therapy may be successful but does not address disease sites outside of the prostate bed/pelvis, and most patients ultimately progress. Nearly all PC over-expresses PSMA; J591 is able to target metastatic disease sites. Full length anti-PSMA mAb has minimal to no access to other sites of low-level PSMA expression. Anti-PSMA-based RIT has demonstrated efficacy, and ^177^Lu is optimal for 1–3 mm (i.e., micrometastatic) lesions.

 Enrollment is ongoing in a multicenter Department of Defense and Prostate Cancer Foundation-sponsored study testing the concept of salvage targeted anti-PSMA-based RIT (http://www.clinicaltrials.gov/ct2/show/NCT00859781/). Men with high-risk CRPC (PSA doubling time <8 months and/or PSA > 20 [73]) and no evidence of disease on CT/MRI and bone scans are randomized in a 2 : 1 fashion to receive double-blinded ^177^Lu-J951 versus ^111^In-J591 (control) with a backbone of hormonal therapy (ketoconazole and hydrocortisone) and will undergo planar gamma camera imaging with SPECT following infusion. The primary endpoint of the study is 18-month metastasis-free survival with additional endpoints of median metastasis-free survival and overall survival. Secondary/exploratory endpoints include evaluation of radiolabeled J591 imaging to detect sites of metastases not apparent on standard CT/MRI and bone scan, validation of adrenal androgen levels as biomarkers for ketoconazole [81], and analysis of circulating tumor cells captured via CellSearch methodology as well as PSMA-GEDI capture [82] for PSMA expression and counts to predict the appearance of radiographic metastases.

## 6. Conclusions

 Biochemical relapse after local therapy for prostate cancer is common. While local salvage therapy is available, deficiencies in imaging currently lead to difficulties in selecting appropriate patients. For those with microscopic sites of disease outside of the prostate/prostate bed, targeted systemic salvage therapy is appealing. Prostate-specific membrane antigen-based diagnostics and therapeutics may lead to improvements in this disease setting.
